# Effects of cone-beam computed tomography with different FOV parameters on simulated internal root resorption volume

**DOI:** 10.1590/1807-3107bor-2025.vol39.088

**Published:** 2025-09-08

**Authors:** Nuray BAĞCI, Arda Büyüksungur, Mehmet Hakan Kurt

**Affiliations:** (a) Gazi University, Faculty of Dentistry, Department of Oral and Maxillofacial Radiology, Ankara, Turkey.; (b) Ankara University, Faculty of Dentistry, Department of Basic Medical Sciences, Ankara, Turkey.; (c) Ankara University, Faculty of Dentistry, Department of Oral and Maxillofacial Radiology, Ankara, Turkey.

**Keywords:** Cone-Beam Computed Tomography, X-Ray Microtomography

## Abstract

The aim of this in-vitro study was to verify which field of view (FOV) in cone-beam computed tomography (CBCT) yields greater accuracy in the detection of internal root resorption (IRR) volume, in comparison to the gold standard of micro-computed tomography (micro-CT) and to a physical method. Twenty-five extractedsingle-rooted teeth were scanned by CBCT with two different FOV parameters (6x6-FOV and 10x10-FOV) and via micro-CT. The volume of dental hard tissue was measured on these images. A simulated IRR was produced by a demineralization protocol. After the simulated IRR, the volumes of the dental hard tissue and the simulated IRR were measured with the same scanning parameters. In addition, the volume of the simulated IRR was measured via a physical method. The simulated IRR volumes obtained by CBCT, micro-CT, and the physical method were statistically compared using one-way ANOVA. Before the simulated IRR, the mean volume of dental hard tissue obtained by 6x6-FOV, 10x10-FOV, and micro-CT were 266.64 ± 11.56, 284.78 ± 14.99, and 233.07 ± 19.91, respectively. The simulated IRR mean volumes obtained by 6x6-FOV, 10x10-FOV, micro-CT, and the physical method were 19.35 ± 5.92, 17.43 ± 5.20, 23.85 ± 6.63, and 13.51 ± 3.11, respectively. The mean volume of the simulated IRR obtained by micro-CT was similar to that of the 6x6-FOV and was significantly different from that of the 10x10-FOV and physical method. The mean volume value of simulated IRR obtained by the physical method was significantly different from those of the micro-CT and 6x6-FOV groups. In conclusion, the 6x6-FOV was better than the 10x10-FOV for the detection of IRR volume by CBCT under clinical conditions.

## Introduction

Cone-beam computed tomography (CBCT) is a three-dimensional (3D) imaging method with high spatial resolution. CBCT has been widely used in dental radiology in recent years for many different indications.^1^ These indications include imaging of osseous lesions, evaluation of dental implants, examination of impacted teeth, analysis of root canal morphology, and detailed examination of root resorption.^2^ CBCT is known to increase diagnostic accuracy and to contribute positively to treatment management.^3^ Scanning parameters affect the resolution and quality of CBCT images.^4^

Volume measurements can easily be performed due to advancements in 3D imaging methods. Specific software-based programs utilizing CBCT data have been developed for volume measurements.^5^ Some of these programs include Dolphin 3D (Dolphin Imaging and Management Solutions, Chatsworth, USA), Mimics (Materialise, Leuven, Belgium), and CTAn (Bruker, Kontich, Belgium).^5-8^ Many studies have been conducted to evaluate the volume of different anatomic structures measured using these programs. Anatomic structures include both large structures, such as the maxillary sinus and nasopharyngeal airways, and small structures such as dental pulp and root resorption.^5-8^ The validation of volume measurements has been reported when high-resolution CBCT images are used.^5^ High-resolution CBCT uses small voxel sizes and is used to visualize small anatomical details and pathologies as well as root fractures, root canals, and root resorption.^9^

Root resorption is a pathology that leads to tooth extraction unless the tooth is treated. Radiological imaging methods are important for the diagnosis of resorption. In particular, 3D imaging methods contribute positively to the diagnosis of resorption because of their advanced features. CBCT images allow both the detection of resorption and its characteristics, such as localization and size.^10^ It can also be used for measuring the volume of resorption.^5^ Underestimation of the resorption volume can lead to progression of resorption, which can lead to root perforation and even tooth extraction.^11^ Overestimation of the resorption volume may lead to tooth extraction without the necessary treatments (such as endodontic treatment). Therefore, accurate determination of the resorption volume is very important for treatment and prognosis under clinical conditions.^5^ Many studies on the detection and measurement of external root resorption volume via CBCT have been reported in the literature.^12-16^ Although many studies have detected internal root resorption (IRR) by CBCT,^17-19^ studies on volume measurement are limited.

To the best of our knowledge, the differences among chemically simulated IRR volumes obtained by CBCT images, micro-computed tomography (micro-CT) images, and physical methods have not been previously investigated. The primary aim of this in vitro study was to verify which field of view (FOV) in CBCT yields greater accuracy in the detection of IRR volume, in comparison to the gold standard of micro-CT. The second aim was to compare the IRR volume obtained by a physical method 3D imaging methods (CBCT and micro-CT). The null hypothesis tested was that there would be no significant difference among the simulated IRR volumes obtained by the different CBCT FOVs, micro-CT, and the physical method.

## Methods

The present study was approved by the Ankara University, Faculty of Dentistry, Clinic Research Ethic Committee (Document Number: 36290600/22/2023) and complied with the Helsinki Declaration. Written informed consent was obtained from all volunteers for the use of the extracted teeth in scientific research.

### Sample preparation

G POWER 3.1.3 program was used to determine the sample size.^20^The minimum sample size to be used for the study was calculated as 25 (95% power level, 95% confidence interval, 1.2 effect size).

**Figure 1:**
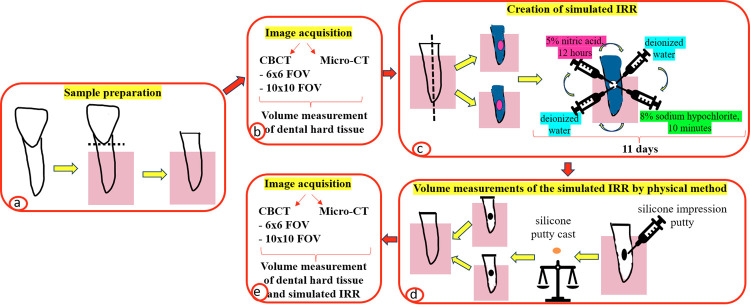
Flowchart of the study. a) Sample preparation: placement into acrylic blocks and separation from the enamel-cement level of 25 single-rooted extracted human teeth; b) image acquisition with CBCT and micro-CT and volume measurement of dental hard tissue; c) creation of simulated IRR: separation in the mesiodistal direction of the sample, production of the 2 identical pieces, and application of demineralization protocols with nitric acid and sodium hypochlorite solutions; d) volume measurements of simulated IRR via the physical method: production of silicone putty casts, weight and volume measurements of the casts, and merging of the 2 identical pieces; e) image acquisition with CBCT and micro-CT and volume measurement of dental hard tissue and the simulated IRR).

In this in vitro study, twenty-five single-rooted human teeth, which were extracted due to periodontal disease, were used. Teeth without caries and restorations were included in the study. All teeth were positioned in acrylic blocks. The crowns and roots of the teeth were separated from the enamel-cementum level using a diamond disk coupled to an electric saw (Micra Cut 201, Metkon Inc., Mauldin, USA). Since the IRR will be simulated in the roots, the root and crown parts of the teeth were separated from each other. Only the roots of the teeth were used. A flowchart of the study method is shown in Figure 1.

### Image acquisition by 3D imaging methods

CBCT and micro-CT were used as 3D imaging methods in this study. During CBCT image acquisition, the 5-pieces block was placed on a table in the form of an occlusal arch. The table was used to simulate a patient undergoing a standing CBCT scan in accordance with the manufacturer’s protocol. Images were acquired using a CBCT device (NewTom Go, Bologna, Italy) with two different FOV parameters (6x6-FOV and 10x10-FOV). The scanning parameters are shown in Table 1. CBCT images of all the samples were acquired.

**Table 1:** Acquisition parameters of the CBCT images.

FOV (cm)	Voxel (mm^3^)	kVp	mA	Time (sec)	DAP (mGy.cm^2^)
6x6	0.150	90	4	5.6	297.86
10x10	0.150	90	4	5.6	720.39

FOV: field of view, kVp: kilovoltage peak, mA: milliamperage, sec: second, DAP: dose area product

Micro-CT images were acquired using a micro-CT device (Skyscan 1172; Bruker-Micro CT, Kontich, Belgium). The scanning protocol included a 20-μm voxel size, 360° rotations in 0.2 steps, 49 milliseconds, and an aluminum filter. Air calibration of the detector was performed before each scan to minimize ring artefacts. During micro-CT image acquisition, the sample were removed from the acrylic block. The acquired projection images were reconstructed with similar parameters for smoothing, ring artefact correction, contrast limits (0.009–0.018), and beam hardening correction (15%) with CTAn software (Bruker, Kontich, Belgium).

The Digital Imaging and Communications in Medicine (DICOM) format of the CBCT and micro-CT images were exported to the CTAn software. The dental hard tissue volume was measured on both the CBCT and micro-CT images.

### Creation of the simulated IRR

The sample teeth were sectioned in the mesiodistal direction with a diamond disk coupled to an electric saw to create the simulated IRR. Therefore, 2 identical pieces were produced. IRR areas of different depths were planned in approximately the middle third and on the inner surface of the 2 identical pieces. Planned IRR areas were covered with wax. Two layers of acid-resistant nail polish were subsequently applied to the other surfaces of the teeth. Thus, the tooth surface outside the planned IRR area was isolated from the acids. The wax was removed from the area after the nail polish dried.

The simulated IRR protocol was performed on these samples as described by Silveira et al.^21^ The simulated IRR was produced by a demineralization protocol using nitric acid and sodium hypochlorite solutions. These solutions were applied to the relevant area with plastic, disposable micropipettes. Deionized water was used to wash the area before each solution was added. Each demineralization cycle consisted of 5% nitric acid solution for 12 hours, deionized water, 8% sodium hypochlorite for 10 minutes, and deionized water. The samples were kept at -1° C (± 3°C) during the entire protocol, which was performed over the course of 11 days. After 11 days, different depth IRR areas were obtained from the samples. Finally, the nail polish was dissolved with a solvent solution.

### Volume measurements of the simulated IRR by a physical method

**Figure 2:**
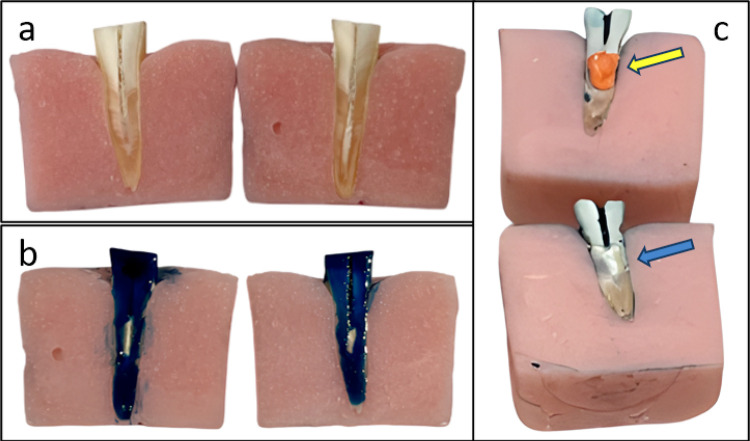
Stages of the simulated IRR. (a: 2 identical pieces; b: nail polish application outside of the planned IRR area; c: silicone putty cast of the simulated IRR (yellow arrow) and the simulated IRR (blue arrow)).

A physical method of measuring simulated IRR volume was applied as described in a previous study.^5^ The simulated IRR areas were moulded using light body silicone impression putty (Elite PP+; Zhermack SpA, Badia Polesine, Italy). The silicone putty was carefully injected directly inside the simulated IRR areas using an automix syringe. At the end of the filling procedure, the identical pieces were repositioned. After the curing time of the silicone putty was completed, putty casts of the simulated IRR areas were obtained (Figure 2).

The weights of the putty casts were measured on a precision scale (RADWAG, Wagi Elektroniczne, Torunska, Radom, Poland). The volumes were calculated by dividing the weight of the simulated IRR cast by the density of the impression putty (d = 1.475 g cc^1^), as described in previous studies.^3,22,23^ The volumes were converted to mm^3^.

### Volume measurements of the simulated IRR by 3D imaging methods

Before scanning, the 2 identical pieces were bonded together with a strong adhesive (Pattex, Henkel, Germany). All the samples were scanned using the same CBCT and micro-CT parameters as before the simulated IRR was created.

**Figure 3:**
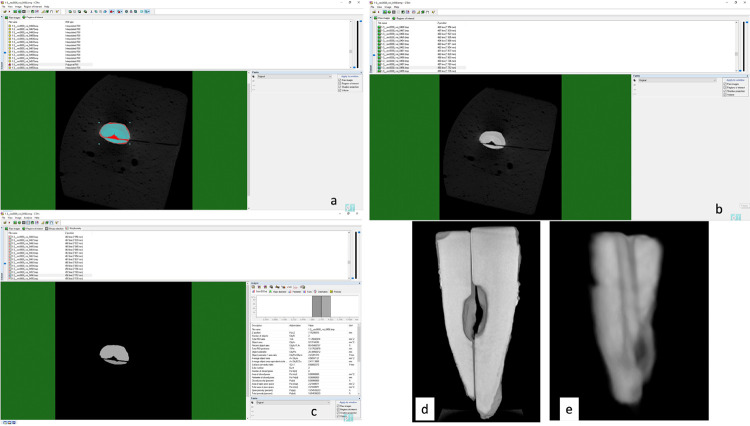
Qualitative analyses of the simulated IRR on micro-CT and CBCT images (a: determination of the region of interest (ROI) on the micro-CT image; b: configuration of the micro-CT images; c: analysis of the micro-CT image; d: the simulated IRR on the micro-CT image; e: the simulated IRR on the CBCT image).

Qualitative analyses of 3D images were conducted to measure the volume of the simulated IRR.^24^ To do this, the structural morphology and boundaries of the simulated IRR areas were determined in detail on 3D images. In this study, the simulated IRR was described as well-circumscribed, oval-shaped resorptive areas on the inner surface of the pulp canal at the level of the middle third of the root.^11^These resorptive areas may or may not extend to the root surface to cause perforation. In addition, these resorptive areas were symmetrical or nonsymmetrical in each of the 2-identical pieces. The volume of dental hard tissue and simulated IRR was measured using the CTAn software on the CBCT and micro-CT images (Figure 3). These measurements were performed by a trained observer (20 years of experience). These measurements were repeated at an interval of 15 days to determine the intraobserver correlation coefficient (ICC).

To determine the degree of hard tissue loss caused by the microcutting effect, 5 sample teeth without IRR were subjected to the same CBCT and micro-CT scanning parameters as those used for the teeth with IRR. The mean amount of hard tissue loss was calculated as a percentage (%). Hard tissue loss was calculated as 7.30%, 6.13%, and 12.63% for 6x6-FOV, 10x10-FOV, and micro-CT, respectively.

### Statistical analysis

Statistical analysis of the data was performed using IBM SPSS Statistic software (IBM SPSS Statistic, USA) Windows version 23.0. Descriptive statistics were performed for minimum-maximum values, means, and standard deviations. The normal distribution of the data was confirmed via the Kolmogrov-Smirnov test. Thus, the volume measurements for dental hard tissue and simulated IRR obtained by CBCT, micro-CT, and the physical method were statistically compared by using one-way ANOVA complemented with Tukey’s test. Intraobserver agreement was assessed by the intraobserver correlation coefficient (ICC) (< 0.50; poor reliability, 0.50-0.75; moderate reliability, 0.75-0.90; good reliability, > 0.90; excellent reliability).^25^ The level of significance was set at 5%.

## Results

Before the simulated IRR was created, the dental hard tissue mean volumes obtained by micro-CT, 6x6-FOV, and 10x10-FOV images were 233.07 ± 19.91, 266.64 ± 11.56, and 284.78 ± 14.99, respectively. The minimum volume of dental hard tissue was measured with micro-CT. There was no statistically significant difference between these groups (Table 2).

**Table 2:** Descriptive statistics and statistical analyses of dental hard tissue volume measurements (mm3) obtained by micro-CT and CBCT before and after the simulated IRR.

Groups	Min	Max	Mean±sd	95%CI		p-value
				Lower bound	Upper bound	
Before the simulated IRR						
Micro-CT	120.13	364.91	233.07 ± 19.91	204.21	261.93	0.071
CBCT						
6x6-FOV	139.55	427.05	266.64 ± 11.56	232.97	300.31	
10x10-FOV	148.64	454.62	284.78 ± 14.99	249.67	319.84	
After the simulated IRR						
Micro-CT	80.10	293.70	179.80 ± 12.57	153.97	205.63	0.086
CBCT						
6x6-FOV	92.54	337.86	207.24 ± 13.41	176.94	237.55	
10x10-FOV	101.35	364.46	225.29 ± 18.80	192.76	257.81	

Micro-CT: micro-computed tomography, CBCT: cone-beam computed tomography, FOV: field of view, min: minimum value, max: maximum value, mean±sd: mean ± standard deviation.

After the simulated IRR was created, the dental hard tissue mean volumes obtained by micro-CT, 6x6-FOV, and 10x10-FOV images were 179.80 ± 12.57, 207.24 ± 13.41, and 225.29 ± 18.80, respectively. The minimum volume of dental hard tissue was measured with micro-CT. However, there was no statistically significant difference between these groups (Table 2).

The simulated IRR mean volumes obtained by micro-CT, 6x6-FOV, 10x10-FOV, and the physical method were 23.85 ± 6.63, 19.35 ± 5.92, 17.43 ± 5.20, and 13.51 ± 3.11, respectively. The minimum volume of the simulated IRR was determined with the physical method, and the maximum volume of the simulated IRR was determined with the micro-CT imaging. The simulated IRR mean volumes were significantly different between these groups (p < 0.000). The mean volume of the simulated IRR obtained by micro-CT was similar to that of the 6x6-FOV and was significantly different from that of the 10x10-FOV and the physical method. The mean volume of the simulated IRR obtained by the physical method was significantly different from those of micro-CT and the 6x6-FOV groups (Table 3).

**Table 3:** Descriptive statistics and statistical analyses of the simulated IRR volume measurements (mm3) obtained by micro-CT, CBCT, and the physical method.

Groups	Min	Max	Mean±sd	95% confidence interval		p-value
				Lower bound	Upper bound	
Micro-CT	3.75	56.81	23.85 ± 6.63^a^	19.46	28.24	0.000*
CBCT						
6x6-FOV	10.13	31.00	19.35 ± 5.92^a^	16.91	21.79	
10x10-FOV	9.10	27.84	17.43 ± 5.20^b^	15.29	19.58	
Physical method	8.25	19.91	13.51 ± 3.11^b^	12.23	13.80	

Micro-CT: micro-computed tomography, CBCT: cone-beam computed tomography, FOV: field of view, min: minimum value, max: maximum value, mean±sd: mean ± standard deviation *statistically significant at p < 0.05, a,b: means followed by different lowercase letters for all groups indicate statistically significant differences in according to ANOVA with the Tukey’s test.

Intraobserver consistency was excellent (ICC = 0.96).

## Discussion

CBCT is a 3D imaging method that best shows the boundaries, size, and localization of IRR areas in clinical conditions. Dentists can use volume measurements from CBCT images for appropriate IRR treatment procedures. In the present study, simulated IRR volume measurements obtained by CBCT with different FOV parameters were compared with those obtained via micro-CT, which is as the gold standard. Additionally, simulated IRR volume measurements obtained by a physical method were compared with the volumes obtained via the 3D imaging methods.

Different CBCT scanning parameters are used in diverse clinical situations. The use of an appropriate scanning parameter is crucial in the diagnosis and treatment of IRR. Several factors affect the selection of an appropriate scanning parameter. These factors can include voxel size, FOV area, observer performance, viewing conditions, and patient-related factors (restored teeth, patient movement, etc.). In a previous study, the diagnostic accuracies of 2 CBCT devices (Iluma Ultra Cone-beam CT Scanner: 3M Imtec, Ardmore, OK, USA and 3D Accuitomo 170: 3D Accuitomo, J Morita Mfg, Corp, Kyoto, Japan) with different voxel resolutions used to detect simulated small internal resorption cavities were compared.^17^ Notably, 0.100 mm^3^-voxel size, 0.200 mm^3^-voxel size, 0.125 mm^3^-voxel size (6x6-FOV), and 0.160 mm^3^-voxel size (8x8-FOV) CBCT images performed similarly and better than 0.300 mm^3^-voxel size in the detection of simulated IRR.^17^ Another study was performed with 2 CBCT devices (I-Cat Next Generation, Imaging Sciences International, Inc., Hatfield, PA and Kodak 900 3D, KODAK Dental System, Carestream Health, Rochester, USA) and 6 different scanning protocols.^5^ The volumes of simulated IRR obtained using the 0.200 mm^3^-voxel size and 0.250 mm^3^-voxel size were significantly greater than those obtained using the 0.300 mm^3^-voxel size in the same device (I-Cat Next Generation).^5^ In addition, the volumes of simulated IRR detected using the 0.076 mm^3^-voxel size and 0.100 mm^3^-voxel size were significantly different than those detected using the 0.200 mm^3^-voxel size in the same device (Kodak 900 3D).^5^ In the present study, the mean volumes of simulated IRR were significantly different for different FOV parameters. The mean volumes for the 10x10-FOV group were greater than those for the 6x6-FOV group. FOV is used in the selection of the scanning area size. Smaller FOV and voxel size values yield better results when measuring IRR volume.^5^ The results of the present study suggest that a smaller FOV provides better measurements of IRR volume. In addition, using a small FOV reduces the effective radiation dose to which the patient is exposed.

Micro-CT has been used as the gold standard in many root resorption studies because it is a nondestructive, high-resolution (< 20 μm voxel size), 3D imaging technique that is sensitive to the detection of small morphological or pathological structures in teeth.^26^ Wang et al. reported that the accuracy of CBCT was very good in detecting external root resorption cavities compared with micro-CT.^13^ Ponder et al. reported that high-resolution (0.200 mm^3^-voxel size) CBCT images were significantly better than low-resolution (0.400 mm^3^-voxel size) CBCT images for volume measurements of external lateral root resorption compared with volume measurements obtained from micro-CT (0.018 mm^3^-voxel size) images.^15^ The present study investigated both large-FOV (10x10-FOV; 0.150 mm^3^-voxel size) and small-FOV (6x6-FOV; 0.150 mm^3^-voxel size) CBCT images for volume measurements of simulated IRR. These volume measurements were compared with those of micro-CT (0.020 mm^3^-voxel size), which is a validated gold standard for the evaluation of hard tissues.^26^ According to the findings of the present study, the mean volumes obtained by CBCT with a 10x10-FOV were significantly smaller than the mean volumes obtained by micro-CT. In addition, there was no significant difference between the mean volumes obtained by CBCT with the 6x6-FOV and those obtained via micro-CT. CBCT images obtained at large FOV values have decreased image quality due to the decrease in resolution. Therefore, the existing IRR volume value may be underestimated. To avoid misestimation, it is important to choose a small FOV instead of a large FOV, especially when visualizing a single tooth.

CBCT scans use ionizing radiation, which has biological risks. Therefore, many in vitro and ex vivo experimental studies have been conducted to optimize the use of CBCT.^5,11,13,14-17,19^ In experimental studies, in vivo conditions and clinical conditions should be accurately simulated. In previous studies, extracted teeth, dry anatomical specimens, and soft tissue simulator materials were used.^5,13,15,17^ In recent years, the effect of different soft tissue simulator materials on radiographic images (such as pixel intensity and image quality) has been investigated.^27-32^ Generally, the simulator materials used do not affect the pixel density of dental tissue in CBCT images.^27,28^ In the present study, we exposed the pulp chamber to create a simulated IRR. According to the user manual of microcut device we used for this procedure, extracted teeth must be placed in moulds (acryl, etc.). We placed the extracted teeth one by one in acrylic moulds it was difficult to use soft tissue simulator material during image acquisition. Thus, we did not use a soft tissue simulator material as was done in other studies.^12,13,17,21^

Most experimental studies on root resorption assessed via CBCT images investigate external root resorption as opposed to IRR.^5,12-19^ Simulation of external root resorption via mechanical methods is preferred over simulation of IRR via chemical methods mainly because it is simple and faster. To create IRR, the pulp chamber must be exposed. In addition, to create resorption with the chemical method, the duration of action of the applied substance should be waited. Kolsuz et al. reported that the visibility on CBCT images of external root resorption created by chemical methods was less than that of external root resorption created by mechanical methods because the cutting edge created by mechanical methods is more easily detected on CBCT images.^14^ On the other hand, chemically induced resorption boundaries have smooth margins and are more similar to resorption boundaries induced in vivo.^32^ However, this makes them difficult to detect. The chemical method developed by Da Silvera et al. was found to be sufficient for simulating IRR and has been used in other studies.^5,21,32^ Since there are a limited number of studies on volume measurements of IRR simulated by chemical method, we used chemical method in the present study.

The volume measurement of the simulated resorption areas obtained by the radiological imaging method and the physical method were compared.^3,5,13,15,33,34^ Both methods have been used for volume measurements of larger defect areas, such as cystic jaw lesions, bone defects, and periapical lesions, as well as smaller defect areas such as external root resorption and IRR.^3,13,15,33,34^ Some studies even refer to the physical method as the gold standard.^5,23^ Because of its low voxel size and high resolution, micro-CT has been validated as the gold standard.^26^ In addition, unlike other studies, the mean volume measurement values obtained by micro-CT and the physical method were compared in the present study. According to our findings, there was a statistically significant difference between the mean volumes simulated IRR obtained by the physical method and those obtained via micro-CT images. There was a statistically significant difference between the mean volumes of simulated IRR obtained by the physical method and those obtained from the CBCT images with a 6x6-FOV. Additionally, the mean volumes of simulated IRR obtained by the physical method were the smallest among all the groups. When the chemical method is used for resorption, there may be an area of decalcification that the imaging method can detect but the physical method cannot. Therefore, there may be differences between the volumes obtained by imaging methods and those obtained via physical methods. We do not recommend the physical method as the gold standard, especially when studying a small area such as in the case of IRR.

This study has several limitations. To simulate IRR, we had to expose the pulp chamber of the teeth via micro-cutting. Some dental hard tissue was lost during this procedure, and it was difficult to determine the amount lost. To overcome this limitation, microcutting was performed on only 5 sample teeth from outside the study group, and these teeth did not have IRR. The same radiologic imaging methods used in the study protocols were applied, and volume calculations were made before and after the procedure. On the basis of these volume calculations, the amount of dental hard tissue lost was obtained as a percentage.

## Conclusion

On the basis of the results presented in this study, it can be concluded that the use of a 6x6-FOV is better than the use of a 10x10-FOV for the detection of IRR volume by CBCT under clinical conditions. Moreover, the simulated IRR volume obtained by the physical method is smaller than that obtained by 3D imaging methods.

## Data Availability

The contents underlying the research text are contained in the manuscript.
